# Artificial intelligence agents as advanced decision support systems in public decision-making: evidence from Peru

**DOI:** 10.3389/frai.2026.1805539

**Published:** 2026-05-13

**Authors:** Emilio J. Medrano-Sánchez, Jadira Jara

**Affiliations:** 1 Faculty of Engineering, Universidad Tecnológica del Perú, Lima, Peru; 2 Graduate School, Universidad Tecnológica del Perú, Lima, Peru

**Keywords:** algorithmic governance, artificial intelligence agents, decision support systems, empirical evidence, public decision-making, public sector

## Abstract

Decision making in the public sector faces persistent challenges that have driven the analysis of technologies aimed at strengthening institutional performance. Within this context, the present study examined the association between the valuation of artificial intelligence agents (AIA) and decision making among public officials with national-level responsibilities in Peru. In this study, AIA are conceptualized as advanced decision support systems (DSS) that complement human judgment in complex public-sector decisions. A Likert-scale survey was administered to 93 participants and showed high internal consistency (Cronbach's alpha = 0.952). Given the non-parametric nature of the data, associations were estimated using Kendall's Tau-b and Spearman's Rho. The results revealed a positive and statistically significant association between the valuation of AIA and overall decision-making (Tau-b = 0.588; Rho = 0.653; *p* < 0.001). Complementarily, the observed pattern was consistent with the notion that higher valuation of AIA is linked to more favorable perceptions of the decision-making process across the analyzed dimensions. In conclusion, the findings support that, in the Peruvian context, the valuation of AIA is associated with decision making perceived as more effective, suggesting their relevance as a complementary support to human judgment and as an AI-based DSS to guide evidence-based modernization of public management.

## 1 Introduction

Decision making in the public sector plays a strategic role in the State's ability to transform resources into effective outcomes; therefore, its strengthening entails quantifiable economic and productive implications. In primary education, internal inefficiency linked to dropouts and grade repetition leads to the waste of 8.1% of public expenditure, amounting to USD 32.6 billion in a single year, which evidences a sustained fiscal loss associated with ineffective public decisions (Mizunoya and Zaw, 2017). In public infrastructure, deficient decision-making processes translate into increasing delays and cost overruns, which doubled in 2021 compared to the previous decade in projects of the United States Army Corps of Engineers, revealing a deterioration in the efficiency of large-scale decisions (Strelzoff et al., 2024). In the European context, evidence shows that larger public contracts and intensive subcontracting are associated with greater inefficiency, while delays decrease when contractual decisions rest with authorities possessing greater institutional experience, underscoring the critical role of organizational decision-making capacity (Caserta et al., 2025).

This problem extends to strategic sectors that are intensive in human and financial resources, confirming its cross-cutting nature within the public sector. In higher education, institutional efficiency varies significantly depending on the type of funding and the surrounding environment, indicating that budgetary decisions directly influence cost and productivity frontiers (Sav, 2012). In public health, inefficient decisions generate persistent macroeconomic impacts, as observed in Indonesia, where the economic cost of smoking ranges from 1.16 to 2.59% of GDP, reflecting substantial losses for the national economy (Meilissa et al., 2022). Similarly, inefficient management of public land is associated with resource waste and low productivity, reinforcing that decision making inefficiency affects long-term public value creation (Grover, 2009).

In light of losses of this magnitude, the contemporary literature questions the adequacy of traditional decision approaches to manage growing volumes of information, time pressure, and institutional complexity, which has driven the exploration of alternatives grounded in quantitative evidence (Alrawahna et al., 2025). In this context, AIA emerge as one of the alternatives examined globally to support public decision making, as error reductions of up to 95% in clinical diagnosis, 80% in medication administration, and 55% in prescribing have been reported, indicating their potential to mitigate critical decision failures (Alqaraleh et al., 2025). Likewise, time savings ranging from 30 to 50% have been documented in radiological screening and specialized review processes, demonstrating their capacity to alleviate operational bottlenecks (Moger et al., 2025). In public administration, studies report a significant positive correlation of up to 60% between the adoption of artificial intelligence (AI) and the speed or efficiency of decision making, suggesting a measurable impact on institutional performance (Dziundziuk et al., 2024). Nevertheless, the evidence also warns that standardized cross-sector metrics remain limited, particularly in governance and ethics dimensions, reinforcing the need to empirically examine their contribution in specific contexts (Agarwal and Nene, 2025; de Almeida and dos Santos, 2025).

Along the same lines, the need for decision efficiency described at the global level is persistently reflected in Latin America through waste, cost overruns, and delays in infrastructure, health, education, and other services, weakening the effectiveness of public spending and eroding citizens' trust, which in turn limits the region's capacity to sustain inclusive development (Yupari and Rodriguez, 2025). At the same time, quantitative evidence is considered essential for identifying root causes and guiding reforms aimed at addressing the technical and structural determinants of these inefficiencies (Yupari and Rodriguez, 2025), within a context characterized by corruption in public procurement (Abramo, 2005), weaknesses in procurement governance (Abramo, 2005), and digital and data gaps (Cedeño and Paz, 2024; Quispe, 2017).

In particular, public infrastructure in Latin America exhibits systematic deviations that reveal shortcomings in decisions related to planning, control, and procurement. In Peru, public works contracts recorded cost increases ranging from 8.8 to 52.2% and time overruns between 4.7 and 524.2%, with estimated economic losses of PEN 19 million attributable to deficiencies in quality and contractual compliance (Romero and Esenarro, 2024). In Colombia, 81.9% of 149 road projects showed schedule or cost deviations (Gómez-Cabrera et al., 2025a), while in educational infrastructure, average deviations of 77.73% in time and 22.17% in costs were reported (Gómez-Cabrera et al., 2025b). In Brazil, procurement mechanisms such as reverse auctions have been shown to encourage risk-taking behavior, increasing contract modifications and cost overruns (Signor et al., 2017), while collusive bidding practices and cartels among construction firms further exacerbate cost overruns (Medeiros-Costa, 2024). Consistently, deficient technical documentation, poor planning, and design errors have been identified as recurrent causes (Yupari and Rodriguez, 2025), together with failures in procurement processes (Cuéllar-Reyes et al., 2023).

This regional pattern cannot be explained solely by technical deficiencies, but also by structural and institutional factors. It has been reported that corruption in public procurement is quantitatively associated with cost overruns and with the non-completion of projects (Abramo, 2005), and that higher levels of competition (more bidders) are linked to lower cost overruns (Pinheiro et al., 2019), whereas collusive bidding practices increase costs (Signor et al., 2017). In addition, electoral years have been shown to correlate with higher cost overruns (Pinheiro et al., 2019), and, in certain contexts, political factors may outweigh technical ones in explaining cost overruns (Catalão et al., 2022). In parallel, gaps in state capacity and regulatory quality have been highlighted as being associated with efficiency gaps (Afonso and Fraga, 2024; Delprato and Antequera, 2021; Pinilla-Rodríguez et al., 2023), while digital and data gaps are reflected in limited impacts of e-government, low digital skills, and poor data management (Afonso and Fraga, 2024), as well as insufficient infrastructure, lack of skills, and inadequate data protection (Cecchini, 2006, 2008), which reduce service inclusion and efficiency (Afonso and Fraga, 2024).

In response to these structural and informational constraints, the literature has increasingly conceptualized artificial intelligence agents as advanced DSS capable of supporting public decision-making. From this perspective, AIA do not replace human judgment, but rather augment it by processing large volumes of data, identifying patterns, and generating structured information to support complex decisions under conditions of uncertainty. This approach situates AIA within the broader tradition of DSS, emphasizing their role as complementary decision support tools that enhance analytical capacity, consistency, and responsiveness in public-sector decision-making.

Within this regional framework, the need for decisional efficiency takes on a concrete expression in the Peruvian public sector, where it manifests through delays, cost overruns, under-execution, and procedural irregularities that affect projects and policies with national impact (Yupari and Rodriguez, 2025). These deficiencies are not attributable solely to operational problems, but are instead associated with institutional weaknesses, limitations in state capacity, and digital and data gaps that condition the quality of decisions adopted at central levels of the State (Escobar et al., 2021; Mamani-Flores et al., 2025).

Quantitative evidence shows that public works contracts experience schedule extensions ranging from 4.7 to 524.2% relative to what was originally planned, reflecting substantial deficiencies in project planning and initial cost estimation (Romero and Esenarro, 2024). Likewise, it has been documented that 67% of construction firms deliver projects behind schedule, generating significant economic impacts associated with inefficient decisions throughout the project life cycle (Lopez-Uchuya et al., 2022). Consistently, deficient technical documentation, poor planning, and inaccurate cost estimates have been identified as the main factors explaining these delays (Yupari and Rodriguez, 2025).

These decisional deficiencies also translate into significant cost overruns, with increases ranging from 8.8 to 52.2% of the contractual value, as well as quantifiable economic losses, such as the PEN 19 million reported in public infrastructure projects affected by contractual non-compliance and quality deficiencies (Romero and Esenarro, 2024; Sánchez et al., 2023). In addition, there is a high incidence of project suspension and underexecution, with an estimated 44% of stalled projects associated with the use of traditional planning and execution methodologies, which limit the ability to anticipate risks and manage decisional complexity (Traverso-Frisancho and Romero-Alva, 2023).

Complementarily, irregularities in public procurement processes and the fragmentation of administrative systems have increased the procedural burden on regulatory bodies, hindering decision traceability and weakening control mechanisms (Urbina and Calderón, 2023; Vílchez et al., 2023). Audit findings reinforce this evidence by revealing persistent economic damages derived from deficient oversight and failures in risk management in public projects (Ipanaque et al., 2024; Sánchez et al., 2023). Taken together, these results confirm that inefficiency in the Peruvian State originates, to a large extent, in decision making processes adopted by officials with national-level responsibility, whose institutional and technical limitations condition the performance of public spending (Escobar et al., 2021).

In continuity with the evidence of decision making inefficiencies described at the global, regional, and national levels, the literature reports a recent expansion in the use of systems based on AIA and Decision Support Systems (DSS) in public decision processes, with outcomes assessed through quantifiable KPIs such as percentage changes, time savings, and error reduction (Kahraman, 2025). Along these lines, a 35% improvement in decision speed has been documented in public and governmental contexts (Abogunrin et al., 2025; Sposato, 2025), while, in specific workflows, reductions of up to 70% in processing time have been reported (Connor et al., 2015). Complementarily, forensic analyses have recorded reductions of up to 70% in processing time (Abogunrin et al., 2025), and in systematic review screening processes, a five- to six-fold reduction in abstract selection time has been observed (Aya et al., 2025), suggesting relevant operational gains in information-intensive tasks.

Regarding decision-making accuracy, the evidence includes direct performance metrics. In experimental and quasi-experimental settings, studies reported a 15%– 17% increase in accuracy with AI support (Lalithadevi et al., 2025; Omar et al., 2025), and observational studies indicated an additional 16%–17% improvement in accuracy, moderated by task complexity (Jeon et al., 2025). In sector-specific applications, finance studies reported 15.9% precision, 16.8% recall, and a 16.7% F1 score (the harmonic mean of precision and recall) for fraud detection (Omar et al., 2025), while education studies reported 12%–20% improvements in student performance and 70% grading efficiency in online learning (Mahamad et al., 2025). In health care, Clinical Decision Support Systems (CDSS) reported 89% (diabetes), 99% (heart disease), and 86% (bone cancer), with an overall accuracy of 91.33% (Lalithadevi et al., 2025), indicating that quantitative measurement of accuracy is available, although dispersed across domains and tasks.

Nevertheless, the literature also reports moderating effects that condition the magnitude of the benefits. Among highly skilled users, studies have shown that they may rely less on AI and, at the same time, report slight decreases in accuracy, even when confidence increases (Peng et al., 2024). In addition, AI literacy has been associated with a greater willingness to adopt the system (Aljuneidi et al., 2024), human self-confidence has influenced the acceptance or rejection of recommendations (Chong et al., 2022), and task complexity has reduced the ability to discern output quality, thereby affecting decision outcomes (Estévez-Almenzar et al., 2025). Taken together, these findings suggest that quantitative outcomes do not depend solely on the tool, but also on the decision-maker's profile and the type of decision involved.

With respect to transparency and auditability, the literature indicates that they remain persistent challenges in public-sector settings (Olsen et al., 2024). It has been noted that explainable AI can improve perceptions of fairness and trust (Carullo, 2023), that algorithmic transparency is often limited and that regulatory frameworks are emerging to address these gaps (Olsen et al., 2024), while public trust could be strengthened through robust ethical frameworks and participatory governance arrangements (Banerjee et al., 2025; Radanliev, 2025). However, in these dimensions, the literature does not report standardized quantitative metrics of transparency or auditability, which restricts direct comparison with empirical results based on numerical indicators.

With regard to consistency and coherence, the literature reports that AIA can enhance both attributes, noting that data-driven models improve consistency, while human judgment remains necessary to mitigate algorithmic biases (Kahraman, 2025; Kostiuk et al., 2025; Peng et al., 2024). It has also been argued that optimal performance emerges when human-AI complementarity operates at high levels (Buschmeyer et al., 2024). Nevertheless, as in the case of transparency, the literature does not provide explicit quantitative measures of coherence or consistency, and therefore their use for numerical comparison should be approached with caution.

Based on the foregoing, an operational knowledge gap is identified: while quantitative evidence exists for improvements in speed and accuracy across multiple domains, the discussion of transparency and coherence remains largely framed in terms of challenges, conditions, and emerging frameworks, without standardized quantitative metrics. Consequently, there remains a need to generate empirical evidence applied to the public sector that allows these dimensions to be examined within a single analytical framework, in line with AI-based DSS approaches, using observable indicators among decision-makers with national-level responsibility.

Consequently, based on the global, regional, and national evidence previously presented regarding quantifiable decision inefficiencies in the public sector, as well as on recent findings suggesting a potential contribution of AIA to key dimensions of the decision process, the following research question is posed: what is the association between the valuation of AIA and decision making among public officials in Peru, considering both the overall quality of the decision process and its dimensions? Specifically, the study seeks to determine how such valuation is associated with the speed of the decision making process, with the perceived accuracy of public decisions, with the integrality of the decision process understood as the incorporation of multiple sources and perspectives, with the transparency of the decision making process, and with the coherence of decisions in relation to institutional normative and strategic frameworks.

Under this logic, and in coherence with a non-experimental and correlational approach, the general objective is to analyze the association between the valuation of AIA and decision making among public officials, considering the overall quality of the decision process and its main dimensions within the context of the Peruvian public administration; more specifically, the study aims to analyze the association between such valuation and the speed of the decision process, the perceived accuracy of decisions, the integrality of the process, the transparency of the decision making process, and the coherence of decisions with institutional normative and strategic frameworks.

Based on the evidence reviewed, the present study is justified by its theoretical contribution to the analysis of public decision making assisted by AIA. It has been argued that AI-based decision making support systems contribute to strengthening governance quality and institutional performance, becoming embedded within principles of adaptive governance that prioritize flexibility, iterative learning, and collaboration among stakeholders (Kurhayadi, 2025). Likewise, frameworks such as the ABCD approach (Artificial Intelligence, Blockchain, Cloud Computing, and Data Analytics) have been proposed to explain how the incorporation of emerging technologies helps address complex challenges and optimize service delivery through data-driven decisions, evidence-based policymaking, predictive analytics, and transparency (Srinivasan et al., 2024). Along the same lines, the link between technological innovation and institutional change has been grounded in public value and stakeholder trust theories, which connect technological adoption with improvements in governance (Bokhari et al., 2025).

Methodologically, the study is justified because the literature identifies that AI-based DSS provide tools for analyzing large volumes of data, detecting patterns, and generating information that is useful for evidence-based decisions (Arora et al., 2024). In addition, the use of systematic reviews and bibliometric analyses has been reported to assess the state of AI adoption in the public sector, identifying readiness dimensions such as institutional capacity, digital infrastructure, regulatory frameworks, and human resource competencies (Gharbaoui et al., 2025; Kurhayadi, 2025). Complementarily, experimental designs such as deliberative surveys have been used to measure public support for AI in governance, with increases in support observed after informed deliberation (Arnesen et al., 2025). Taken together, these antecedents support the relevance of producing applied empirical evidence that links the valuation of AIA with observable dimensions of the decision making process in the public sector (Arora et al., 2024).

From a practical perspective, it has been indicated that AI-based DSS can enhance the quality of governance by improving operational efficiency, service delivery, and decision accuracy through the automation of administrative processes, optimization of resource allocation, and real-time feedback enabled by tools such as chatbots and sentiment analysis (Bendary et al., 2026). Likewise, it has been suggested that data-driven decision making enabled by AI may contribute to the Sustainable Development Goals (SDGs) by improving resource management and public policy implementation (Kumar et al., 2025; Siripipatthanakul et al., 2025). Along these lines, it has been argued that the automation of routine tasks and the use of predictive information make it possible to anticipate future scenarios and develop proactive strategies in public management (Arora et al., 2024; Kumar et al., 2025). Nevertheless, it has also been noted that practical challenges related to algorithmic bias, transparency deficits, and ethical dilemmas persist, thereby requiring robust governance frameworks and ethical guidelines to ensure responsible adoption (Alrawahna et al., 2025; Rakšnys et al., 2025).

Likewise, the study aligns with the SDGs by contributing to the strengthening of effective, accountable, and transparent institutions, in line with SDG 16. Complementarily, the emphasis on the use of digital technologies and intelligent systems to support public decision making is linked to SDG 9, as it promotes innovation, the modernization of institutional infrastructure, and the strategic use of data for policy formulation. Finally, the analysis of decision making from a systemic and cooperative perspective is related to SDG 17, by highlighting the need to strengthen capacities, interinstitutional coordination, and partnerships to ensure the effective implementation of technological solutions in the public sector.

## 2 Methodology

The study was designed with the purpose of analyzing the relationship between AIA and decision making within the field of public management, which required the adoption of a methodological approach that allowed for the empirical examination of public officials' perceptions based on structured measurements. In this regard, a quantitative approach was selected, as it enabled the systematic collection of numerical data and their subsequent analysis through statistical techniques. This approach was appropriate for exploring associations between the variables of interest without intervening in or manipulating the observed phenomena.

Under this logic, the research was conducted with a basic scope, oriented toward the generation of empirical and theoretical knowledge regarding the use of AIA in public sector decision making processes. The study was not intended for the immediate application of its results, but rather to contribute to the academic understanding of an emerging phenomenon from an analytical perspective. Likewise, a non-experimental, cross-sectional design was adopted, given that the variables were observed in their natural state and data collection was carried out at a single point in time, allowing for the description of their characteristics and the identification of associations between them, without establishing causal relationships.

In order to ensure the internal coherence of the study, a consistency matrix was developed (Supplementary File 1), which allowed for the systematic articulation of the research problem, the general and specific objectives, the hypotheses, the variables, as well as their dimensions and indicators. This methodological instrument facilitated the alignment between the conceptual framework and the empirical design and served as the basis for the construction of the questionnaire and the definition of the analytical criteria. Complementarily, a variable operationalization matrix was developed, detailing the conceptual and operational definitions of AIA and Decision Making, as well as the indicators and items. This instrument enabled the translation of theoretical constructs into concrete and quantifiable measures (Supplementary File 2).

Regarding the study population, it was composed of public officials and civil servants who performed functions involving institutional decision authority, excluding those who did not directly participate in decision processes. From this population, a sample of 93 participants was selected through non-probabilistic purposive sampling. This sample size is consistent with prior survey-based research in comparable applied research contexts (Medrano-Sánchez et al., 2025; Medrano-Sánchez and Ochoa-Tataje, 2024). This criterion responded to the need to access respondents with direct experience in decision making within the public sector, prioritizing the relevance of the information over statistical representativeness. Although this type of sampling limits the generalizability of the results, it allowed for the collection of relevant and context-specific data for analyzing the associations between the variables under study.

Data collection was conducted through the administration of a structured survey consisting of 27 items with closed-ended responses, organized using a five-point Likert scale. The items were developed based on the indicators defined in the consistency matrix and covered the dimensions corresponding to the AIA variable and the decision making variable. The survey was administered virtually through the QuestionPro platform, which facilitated instrument distribution and automated data capture. The Spanish version of the survey (Supplementary File 3), as well as its English version (Supplementary File 4), have been included as Supplementary material.

Instrument validity was assessed through expert judgment, whereby specialists reviewed the coherence, clarity, and relevance of the items in relation to the variables and dimensions analyzed. Reliability, in turn, was determined using the Cronbach's alpha coefficient, obtaining a value of 0.952, which indicated a high level of internal consistency of the instrument. Statistical analyses were conducted using SPSS software, version 29.0.2.0.

For data analysis, an initial descriptive stage was conducted to examine the joint distribution of the variables and their dimensions. For this purpose, cross-tabulation tables were constructed, allowing the identification of frequency patterns, percentage concentrations, and general trends in the valuation of AIA and across the different dimensions of decision making. This descriptive analysis provided an initial approximation to the behavior of the data and facilitated the understanding of the observed relationships from an exploratory perspective.

Subsequently, inferential data analysis was conducted. First, the Kolmogorov–Smirnov test was applied to assess the normality of the distribution. Given that the results indicated a non-normal distribution, Spearman's Rho and Kendall's Tau-b correlation coefficients were employed, as they were appropriate considering the measurement level of the variables and the non-experimental design of the study. This analysis made it possible to examine the association between AIA and decision making without inferring causal relationships.

Finally, the administration of the instrument was conducted under strict ethical considerations. Prior to starting the survey, participants were presented with an informational screen explaining the purpose of the study, the absence of personal data collection, and the guarantee of confidentiality and anonymity of responses. Participation was voluntary, and informed consent was obtained from all participants before completing the survey, with the option to withdraw at any time without any consequences. The collected responses were stored on encrypted servers with access restricted exclusively to the researcher. In addition, periodic backups were performed to ensure data integrity. Given that the study did not involve any intervention, experimentation, or collection of sensitive personal data, and was based solely on voluntary perception surveys, it was classified, according to institutional ethical guidelines, as exempt from formal review by an ethics committee. in accordance with institutional research and ethics guidelines applicable to non-interventional studies.

## 3 Results

This section presents the results derived from the analysis of the information collected through the survey administered to public officials with decision-making authority. First, descriptive results are reported, which made it possible to examine the distribution and joint behavior of the AIA and decision making variables, as well as each of the dimensions that compose the latter. Subsequently, the section presents the inferential results aimed at testing the proposed hypotheses.

### 3.1 Descriptive results

In relation to the general objective of the study, Table 1 presents the joint distribution between the assessment of AIA and decision making. The descriptive analysis showed that when AIA were rated as poor (37.6% of the sample), poor decision making predominated, accounting for 26.9% of total responses, while only 2.2% associated this technological assessment with good Decision Making. By contrast, when AIA were rated at a fair level (30.1%), the distribution of decision making exhibited a more balanced pattern, with similar proportions across the poor, fair, and good categories. Finally, when AIA were assessed as good (32.3%), the poor decision making category was absent, and a clear shift toward favorable assessments was observed, primarily concentrated in the good decision making category (19.4%). This descriptive pattern suggests that higher valuations of AIA are associated with more favorable perceptions of overall decision-making performance.

**Table 1:** Cross-tabulation: AIA and decision making.

Independent variable	Level	Measure	Decision making			
			Poor	Fair	Good	Total
AIA	Poor	Count	25	8	2	35
		% of total	26.9%	8.6%	2.2%	37.6%
	Fair	Count	8	12	8	28
		% of total	8.6%	12.9%	8.6%	30.1%
	Good	Count	0	12	18	30
		% of total	0.0%	12.9%	19.4%	32.3%
Total		Count	33	32	28	93
		% of total	35.5%	34.4%	30.1%	100.0%

In relation to specific objective 1, Table 2 examines the association between the assessment of AIA and the speed dimension of decision making. The descriptive results showed that when AIA were rated as poor (37.6%), a negative perception of speed predominated, accounting for 29.0% of total responses. When AIA were placed at a fair level (30.1%), perceptions of speed were more evenly distributed across the three categories. In turn, when AIA were assessed as good (32.3%), the proportion of poor speed evaluations decreased substantially, while favorable perceptions of speed increased (18.3%). This percentage shift indicates that higher valuations of AIA are associated with more favorable perceptions of the speed of the decision making process.

**Table 2:** Cross-tabulation: AIA and speed.

Independent variable	Level	Measure	Speed			
			Poor	Fair	Good	Total
AIA	Poor	Count	27	6	2	35
		% of total	29.0%	6.5%	2.2%	37.6%
	Fair	Count	16	6	6	28
		% of total	17.2%	6.5%	6.5%	30.1%
	Good	Count	3	10	17	30
		% of total	3.2%	10.8%	18.3%	32.3%
Total		Count	46	22	25	93
		% of total	49.5%	23.7%	26.9%	100.0%

With regard to specific objective 2, Table 3 presents the descriptive relationship between the assessment of AIA and the accuracy dimension of decision making. The results showed that when AIA were rated as poor (37.6%), a negative perception of accuracy prevailed, accounting for 25.8% of total responses. At the fair level of AIA (30.1%), perceptions of accuracy were more evenly distributed across the poor, fair, and good categories. By contrast, when AIA were considered good (32.3%), an increase was observed in the proportion of good accuracy evaluations (20.4%), along with a reduction in negative perceptions. This descriptive pattern suggests that greater confidence in AIA is associated with more positive perceptions of the accuracy of decision making.

**Table 3:** Cross-tabulation: AIA and accuracy.

Independent variable	Level	Measure	Accuracy			
			Poor	Fair	Good	Total
AIA	Poor	Count	24	8	3	35
		% of total	25.8%	8.6%	3.2%	37.6%
	Fair	Count	10	13	5	28
		% of total	10.8%	14.0%	5.4%	30.1%
	Good	Count	5	6	19	30
		% of total	5.4%	6.5%	20.4%	32.3%
Total		Count	39	27	27	93
		% of total	41.9%	29.0%	29.0%	100.0%

With regard to specific objective 3, Table 4 analyzes the association between the assessment of AIA and the integrality dimension of Decision Making. The data showed that when AIA were rated as poor (37.6%), a negative perception of integrality predominated, accounting for 30.1% of total responses. When AIA were positioned at a fair level (30.1%), perceptions became more diversified, showing greater dispersion across categories. By contrast, when AIA were rated as good (32.3%), a significant reduction was observed in negative perceptions of integrality, along with an increase in positive evaluations. This percentage shift indicates that a more favorable assessment of AIA is associated with more positive perceptions regarding the ability to integrate multiple sources and perspectives in decision making.

**Table 4:** Cross-tabulation: AIA and integrity.

Independent variable	Level	Measure	Integrity			
			Poor	Fair	Good	Total
AIA	Poor	Count	28	4	3	35
		% of total	30.1%	4.3%	3.2%	37.6%
	Fair	Count	18	4	6	28
		% of total	19.4%	4.3%	6.5%	30.1%
	Good	Count	11	4	15	30
		% of total	11.8%	4.3%	16.1%	32.3%
Total		Count	57	12	24	93
		% of total	61.3%	12.9%	25.8%	100.0%

With regard to specific objective 4, Table 5 examines the association between the assessment of AIA and the transparency dimension of decision making. The descriptive analysis revealed that when AIA were evaluated as poor (37.6%), a negative perception of transparency predominated, accounting for 32.3% of total responses. When AIA were positioned at a fair level (30.1%), the distribution of perceptions began to diversify, showing a greater presence of fair and good evaluations. In the case of AIA rated as good (32.3%), a reduction in negative perceptions and an increase in positive assessments of transparency were observed. This pattern suggests that a more favorable assessment of AIA is associated with more positive perceptions regarding the clarity and traceability of the decision-making process.

**Table 5:** Cross-tabulation: AIA and transparency.

Independent variable	Level	Measure	Transparency			
			Poor	Fair	Good	Total
AIA	Poor	Count	30	4	1	35
		% of total	32.3%	4.3%	1.1%	37.6%
	Fair	Count	17	6	5	28
		% of total	18.3%	6.5%	5.4%	30.1%
	Good	Count	9	6	15	30
		% of total	9.7%	6.5%	16.1%	32.3%
Total		Count	56	16	21	93
		% of total	60.2%	17.2%	22.6%	100.0%

With regard to specific objective 5, Table 6 presents the descriptive association between the assessment of AIA and the coherence dimension of Decision Making. The results showed that when AIA were rated as poor (37.6%), a negative perception of coherence predominated, accounting for 25.8% of total responses. When AIA were assessed as fair (30.1%), perceptions were distributed more evenly, whereas when they were considered good (32.3%), a progressive increase in positive assessments of coherence was observed (18.3%). This strictly descriptive percentage shift indicates that a more favorable assessment of AIA is associated with more positive perceptions regarding the alignment of decisions with strategic plans and institutional regulations.

**Table 6:** Cross-tabulation: AIA and coherence.

Independent variable	Level	Measure	Coherence			
			Poor	Fair	Good	Total
AIA	Poor	Count	24	8	3	35
		% of total	25.8%	8.6%	3.2%	37.6%
	Fair	Count	5	16	7	28
		% of total	5.4%	17.2%	7.5%	30.1%
	Good	Count	3	10	17	30
		% of total	3.2%	10.8%	18.3%	32.3%
Total		Count	32	34	27	93
		% of total	34.4%	36.6%	29.0%	100.0%

Overall, the descriptive results revealed a consistent pattern: as the assessment of AIA shifted from lower to higher levels, a progressive movement of perceptions from negative categories toward more favorable ones was observed, both in overall decision-making and across each of its dimensions. This descriptive pattern provides a solid empirical basis for the subsequent inferential analysis of the statistical relationship between both variables.

### 3.2 Inferential results

The inferential results aimed to examine the existence and strength of statistical associations between AIA and Decision Making, as well as between AIA and each of the dimensions that make up Decision Making. To this end, a sequential procedure was followed that first involved assessing data normality and subsequently testing the general and specific hypotheses using correlation coefficients appropriate for ordinal variables. This approach ensured methodological coherence between the study design, the nature of the data, and the statistical techniques employed.

#### 3.2.1 Normality test

To determine the distributional behavior of the data and define the most appropriate inferential tests, normality tests were applied to the AIA and decision making variables. Because the sample size was greater than 50 participants (*n* = 93), the Kolmogorov–Smirnov test was used as the primary evaluation criterion, complemented by the Shapiro–Wilk test, with results shown in Table 7.

**Table 7:** Normality tests.

Variable	Kolmogorov–Smirnov^a^			Shapiro–Wilk		
	Statistic	df	Sig.	Statistic	df	Sig.
AIA	0.087	93	0.076	0.972	93	0.042
Decision making	0.097	93	0.030	0.968	93	0.022

^a^Lilliefors significance correction.

The obtained significance values indicated that, for the AIA variable, the Kolmogorov–Smirnov statistic did not allow rejection of the normality hypothesis (*p* = 0.076), whereas the Shapiro–Wilk test yielded a significance value below 0.05 (*p* = 0.042). In the case of the decision making variable, both tests produced significance values below the 0.05 threshold (*p* = 0.030 for Kolmogorov–Smirnov and *p* = 0.022 for Shapiro–Wilk), indicating a non-normal distribution.

Considering these results and taking into account the ordinal nature of the variables derived from categorized scales, non-parametric statistics were selected for the inferential analysis. Accordingly, Kendall's Tau-b and Spearman's Rho coefficients were used to examine the associations between AIA and decision making, as well as between AIA and each of the analyzed dimensions.

#### 3.2.2 Hypothesis testing

To test the general hypothesis, Kendall's Tau-b and Spearman's Rho coefficients were applied in order to evaluate the association between the valuation of AIA and Decision Making. The consolidated results are presented in Table 8. Both coefficients showed positive and statistically significant values, with two-tailed significance levels below 0.05 (*p* < 0.001). This result allowed the null hypothesis to be rejected and the alternative hypothesis to be accepted, indicating the existence of a statistically significant association between AIA and decision making.

**Table 8:** Correlation results between AIA and decision making.

Correlation type	Independent variable	Measure	AIA	Decision making
Kendall's Tau-b	AIA	Correlation coefficient	1.000	0.588^**^
		Sig. (2-tailed)		< .001
		*N*	93	93
Spearman's Rho	AIA	Correlation coefficient	1.000	0.653^**^
		Sig. (2-tailed)		< .001
		*N*	93	93

^**^Correlation is significant at the 0.01 level (two-tailed).

Regarding the strength of the relationship, Kendall's Tau-b coefficient reached a value of 0.588 and Spearman's Rho a value of 0.653, reflecting a positive association of moderate to high magnitude. From an interpretative perspective, these values indicated that a higher valuation of AIA performance was associated with more favorable assessments of decision making, without implying a causal relationship.

Subsequently, specific hypothesis 1 was tested, referring to the association between AIA and the speed dimension of Decision Making. The results presented in Table 9 showed bilateral significance levels below 0.05 for both coefficients (*p* < 0.001), which allowed rejection of the null hypothesis. In turn, the values of Kendall's Tau-b (0.518) and Spearman's Rho (0.579) indicated a positive association of moderate magnitude, suggesting that higher valuations of AIA were associated with more favorable perceptions of the speed of the decision-making process.

**Table 9:** Correlation results between AIA and speed.

Correlation type	Independent variable	Measure	AIA	Speed
Kendall's Tau-b	AIA	Correlation coefficient	1.000	0.518^**^
		Sig. (2-tailed)		< 0.001
		*N*	93	93
Spearman's Rho	AIA	Correlation coefficient	1.000	0.579^**^
		Sig. (2-tailed)		< 0.001
		*N*	93	93

^**^Correlation is significant at the 0.01 level (two-tailed).

With regard to specific hypothesis 2, the association between AIA and the accuracy dimension of decision making was examined. According to the results reported in Table 10, both coefficients showed levels of statistical significance below the 0.05 threshold (*p* < 0.001). In addition, the values obtained for Kendall's Tau-b (0.483) and Spearman's Rho (0.526) reflected a positive association of moderate magnitude. These results indicated that a higher valuation of AIA was associated with more favorable perceptions regarding the accuracy with which information was selected and analyzed in decision-making processes.

**Table 10:** Correlation results between AIA and accuracy.

Correlation type	Independent variable	Measure	AIA	Accuracy
Kendall's Tau-b	AIA	Correlation coefficient	1.000	0.483^**^
		Sig. (2-tailed)		< 0.001
		*N*	93	93
Spearman's Rho	AIA	Correlation coefficient	1.000	0.526^**^
		Sig. (2-tailed)		< 0.001
		*N*	93	93

^**^Correlation is significant at the 0.01 level (two-tailed).

With regard to specific hypothesis 3, the association between AIA and the integrality dimension of decision making was analyzed, with the results presented in Table 11. The levels of bilateral significance were below 0.05 for both coefficients (*p* < 0.001), which allowed the null hypothesis to be rejected. In terms of magnitude, Kendall's Tau-b reached a value of 0.362 and Spearman's Rho a value of 0.397, evidencing a positive association of low to moderate magnitude. This finding suggested that a higher valuation of AIA was related to a greater inclusion of diverse sources and perspectives during the decision-making process.

**Table 11:** Correlation results between AIA and integrity.

Correlation type	Independent variable	Measure	AIA	Integrity
Kendall's Tau-b	AIA	Correlation coefficient	1.000	0.362^**^
		Sig. (2-tailed)		< 0.001
		*N*	93	93
Spearman's Rho	AIA	Correlation coefficient	1.000	0.397^**^
		Sig. (2-tailed)		< 0.001
		*N*	93	93

^**^Correlation is significant at the 0.01 level (two-tailed).

For the testing of specific hypothesis 4, the association between AIA and the transparency dimension of decision making was examined. The results presented in Table 12 showed bilateral significance levels below 0.05 for both coefficients (*p* < 0.001). The values of Kendall's Tau-b (0.463) and Spearman's Rho (0.508) indicated a positive association of moderate magnitude. From a substantive perspective, these results indicated that more favorable valuations of AIA were associated with more positive perceptions of clarity, traceability, and accountability in decision-making processes.

**Table 12:** Correlation results between AIA and transparency.

Correlation type	Independent variable	Measure	AIA	Transparency
Kendall's Tau-b	AIA	Correlation coefficient	1.000	0.463^**^
		Sig. (2-tailed)		< 0.001
		*N*	93	93
Spearman's Rho	AIA	Correlation coefficient	1.000	0.508^**^
		Sig. (2-tailed)		< 0.001
		*N*	93	93

^**^Correlation is significant at the 0.01 level (two-tailed).

Finally, specific hypothesis 5 was tested, referring to the association between AIA and the coherence dimension of Decision Making. The results reported in Table 13 showed bilateral significance levels below the 0.05 threshold for both coefficients (*p* < 0.001), which allowed the null hypothesis to be rejected. In terms of strength, Kendall's Tau-b reached a value of 0.521 and Spearman's Rho a value of 0.567, reflecting a positive association of moderate magnitude. This result indicated that a more favorable valuation of AIA was linked to more favorable judgments regarding the normative and strategic coherence of the decisions adopted.

**Table 13:** Correlation results between AIA and coherence.

Correlation type	Independent variable	Measure	AIA	Coherence
Kendall's Tau-b	AIA	Correlation coefficient	1.000	0.521^**^
		Sig. (2-tailed)		< 0.001
		*N*	93	93
Spearman's Rho	AIA	Correlation coefficient	1.000	0.567^**^
		Sig. (2-tailed)		< 0.001
		*N*	93	93

^**^Correlation is significant at the 0.01 level (two-tailed).

Overall, the inferential results showed a consistent pattern: across all tests conducted, significance levels were below 0.05 and the association coefficients remained within positive ranges, evidencing statistically significant associations between AIA and Decision Making, both at the global level and across each of its dimensions. These findings reinforced, from a statistical perspective, the trend observed in the descriptive analysis and provided a solid empirical basis for the development of the discussion of results.

## 4 Discussion of results

The results show a positive and statistically significant association between the valuation of AIA and decision making among public officials, with a Kendall's Tau-b coefficient of 0.588 and a Spearman's Rho of 0.653 (*p* < 0.001), indicating a relationship of moderate to high magnitude. This empirical evidence is consistent with studies reporting quantifiable increases in the speed and efficiency of decision making processes when AI-based decision support systems are incorporated, reinforcing the conceptualization of AIA as advanced DSS that support complex public-sector decision processes with improvements of approximately 35% in decision speed in governmental contexts (Abogunrin et al., 2025). Complementarily, it has been documented that AI-related analytical automation enables reductions of up to 70% in operational processing times, reinforcing the notion that higher valuation of these technologies is associated with more agile decision making processes (Connor et al., 2015). Likewise, the correlation observed in the present study is consistent with findings showing positive associations between AI adoption and institutional performance, with coefficients reaching values close to 60% in previous correlational studies within the public sector (Dziundziuk et al., 2024). Nevertheless, other authors caution that these associations do not imply direct causality and depend on organizational conditions, human capacities, and governance frameworks, which aligns with the non-experimental nature of the design and with a cautious interpretation of the coefficients obtained (Agarwal and Nene, 2025). Overall, the empirical evidence from this study confirms, in line with the literature, that higher valuation of AIA is associated with a more favorable perception of Decision Making, without implying causal effects.

In line with the global association identified between the valuation of AIA and Decision Making, the specific results confirm that this relationship is consistently expressed in the “speed” dimension of the decision making process. Specifically, a positive and statistically significant association was observed between the valuation of AIA and speed, with a Kendall's Tau-b of 0.518 and a Spearman's Rho of 0.579 (*p* < 0.001), indicating a relationship of moderate magnitude. This empirical finding is consistent with studies documenting quantifiable increases in decision making speed following the incorporation of AI-based systems, with reported improvements of approximately 35% in public and administrative contexts (Abogunrin et al., 2025). Complementarily, it has been shown that analytical automation enables reductions of up to 70% in operational processing times, particularly in tasks intensive in information analysis, reinforcing the observed association between AIA valuation and greater decision making speed (Connor et al., 2015). Likewise, research on review and information screening processes reports time reductions of five to six times compared to traditional methods, suggesting that speed-related benefits are amplified when decisions require processing large volumes of data (Aya et al., 2025). Nevertheless, other authors note that these improvements do not occur uniformly and depend on the degree of decision structuring and on the decision maker's capacity to interpret and use algorithmic recommendations, introducing variability in the observed effects (Peng et al., 2024). Overall, the evidence confirms that the global association between AIA and decision making identified in this study materializes, in a specific manner, in more favorable perceptions regarding the speed of the decision making process, without implying automatic effects or effects independent of the institutional context.

Beyond the effects observed in the speed of the decision making process, the results indicate that the positive association between the valuation of AIA and decision making is also consistently manifested in the “accuracy” dimension. Specifically, a positive and statistically significant association was identified between the valuation of AIA and the accuracy of the decision making process, with a Kendall's Tau-b of 0.483 and a Spearman's Rho of 0.526 (*p* < 0.001), reflecting a relationship of moderate magnitude. This empirical finding is consistent with studies reporting quantifiable gains in decision accuracy when AI-assisted systems are used, with improvements on the order of 15%–17% compared to decisions made without algorithmic support (Lalithadevi et al., 2025; Omar et al., 2025). Complementarily, observational studies have documented an additional 16%–17% improvement in accuracy, moderated by task complexity (Jeon et al., 2025). Nevertheless, other authors caution that among highly expert decision makers, algorithmic assistance may produce marginal effects or even slight decreases in accuracy due to overconfidence or selective reliance on the system (Peng et al., 2024). Overall, the evidence from the present study confirms that the global association between AIA and decision making is also expressed in more favorable perceptions regarding the accuracy of the decision making process, in line with literature highlighting quantifiable benefits that are nonetheless conditioned by the decision maker's profile and the nature of the task.

After establishing that the valuation of AIA is consistently associated with the speed and accuracy of the decision making process, the results show that this relationship also extends to the “integrality” dimension, although with comparatively lower intensity. Specifically, a positive and statistically significant association was identified between the valuation of AIA and the integrality of Decision Making, with a Kendall's Tau-b of 0.362 and a Spearman's Rho of 0.397 (*p* < 0.001), reflecting a relationship of low to moderate magnitude. This empirical result is consistent, although not directly comparable in numerical terms, with literature suggesting that AI-based systems can broaden the informational scope considered in decision making processes by integrating large volumes of heterogeneous data and multiple analytical criteria, thereby fostering more integrative decisions from a procedural perspective (Kahraman, 2025). Complementarily, data-driven models have been shown to contribute to greater consistency and integration of information, provided that adequate interaction exists between the human decision maker and the algorithmic system (Kostiuk et al., 2025; Peng et al., 2024). Nevertheless, unlike what has been observed for speed and accuracy, the reviewed literature does not report standardized quantitative metrics that allow for a direct measurement of decision making integrality, which limits the possibility of numerically contrasting the coefficients obtained in the present study with prior empirical values. In this sense, the findings reported here provide original quantitative evidence for a dimension that, in previous studies, has been addressed mainly through conceptual or qualitative approaches, emphasizing conditions of human-AI complementarity rather than measurable outcomes (Buschmeyer et al., 2024). Overall, the low to moderate positive association identified suggests that a higher valuation of AIA is linked to more favorable perceptions regarding the integrality of the decision making process, although with less intense effects than those observed for speed and accuracy. This pattern is consistent with literature warning that the benefits of AI in complex judgment dimensions, such as the integration of diverse perspectives, depend to a greater extent on the institutional context, the decision maker's profile, and the quality of human-algorithm interaction, reinforcing the need to interpret these results cautiously and without causal inferences.

In continuity with the findings observed for the dimensions of speed, accuracy, and integrality, the study results show that the positive association between the valuation of AIA and decision making is also consistently expressed in the “transparency” dimension of the decision making process. Specifically, a positive and statistically significant association was identified, with a Kendall's Tau-b coefficient of 0.463 and a Spearman's Rho of 0.508 (*p* < 0.001), indicating a relationship of moderate magnitude between the valuation of AIA and more favorable perceptions regarding clarity, traceability, and accountability in public decision making processes. This empirical result converges with literature arguing that transparency and auditability remain structural challenges in public sector environments, even under conditions of advanced technological adoption (Olsen et al., 2024). Along these lines, explainable artificial intelligence approaches have been shown to improve perceptions of fairness and trust in decision making processes by facilitating understanding of the criteria underlying algorithmic recommendations (Carullo, 2023). Complementarily, it has been documented that algorithmic transparency is often limited and that, in response, regulatory frameworks are emerging to address gaps in traceability and institutional control (Olsen et al., 2024). Likewise, other studies suggest that public trust in AI-assisted decision systems could be strengthened through robust ethical frameworks and participatory governance schemes, which would act as compensatory mechanisms in the face of the technical opacity inherent in certain algorithmic models (Banerjee et al., 2025; Radanliev, 2025). In this sense, the moderate association identified in the present study is consistent with literature recognizing potential benefits in terms of perceived transparency, albeit conditioned by the institutional and regulatory design accompanying the implementation of these technologies.

In continuity with the associations observed for the dimensions of speed, accuracy, integrality, and transparency, the results related to “coherence” show that the valuation of AIA is positively and statistically significantly associated with coherence in Decision Making, with Kendall's Tau-b coefficients of 0.521 and Spearman's Rho of 0.567 (*p* < 0.001), reflecting a relationship of moderate magnitude. This empirical finding is consistent with literature arguing that AI-based systems can contribute to improving decision coherence and consistency by reducing variability across similar decisions through the use of data-driven models (Kahraman, 2025; Kostiuk et al., 2025). Likewise, it has been noted that these systems promote alignment of decisions with predefined normative and strategic criteria, although human judgment remains a necessary component to mitigate algorithmic bias and to contextualize automated recommendations (Peng et al., 2024). Complementarily, the moderate association identified in this study aligns with arguments emphasizing that optimal performance in terms of decision coherence emerges when there is effective complementarity between humans and AI systems, rather than a substitution of human judgment (Buschmeyer et al., 2024). Nevertheless, as also reported in the state of the art, the reviewed literature does not provide explicit and standardized quantitative metrics to measure decision coherence or consistency, which limits direct numerical comparison across studies.

Overall, the discussion shows that the empirical results obtained engage consistently with the specialized literature. In particular, the findings provide empirical support for understanding AIA as AI-based decision support systems, insofar as higher valuation is systematically associated with more favorable perceptions across multiple decision-making dimensions, both in areas where comparable quantitative metrics exist and in dimensions where prior knowledge has been predominantly conceptual. The observed associations reinforce the idea that the valuation of AIA is related to different facets of the decision-making process, albeit with differentiated intensities depending on the nature of each dimension. At the same time, comparison with previous studies highlights that the effects associated with AIA do not manifest in a homogeneous or independent manner, but are mediated by organizational conditions, decision-maker profiles, and institutional frameworks, as emphasized in the literature. In this way, the discussion situates the findings within the current state of knowledge, acknowledging clear convergences, persistent gaps, and interpretative boundaries, without extrapolating the results beyond the empirical scope of the study.

## 5 Conclusion

Based on the empirical evidence obtained, this study concludes that the valuation of AIA is positively and statistically significantly associated with decision making among public officials in the context of Peruvian public administration. This relationship is consistently observed at the overall level of the decision-making process and is expressed, with varying intensity, across the dimensions of speed, accuracy, integrality, transparency, and coherence, suggesting that higher valuation of AIA is linked to more favorable perceptions regarding the efficiency, quality, and institutional alignment of public decisions. Taken together, the findings confirm that AIA constitute a relevant factor in shaping decision-making processes that are more agile, accurate, and institutionally consistent, without implying automatic or causal effects, given the non-experimental nature of the study. In this way, the research provides integrated empirical evidence on the role of AIA in public sector Decision Making, contributing to a field in which fragmented, or primarily conceptual approaches have predominated to date.

## 6 Limitations and future research

### 6.1 Limitations

The findings of this study should be interpreted in light of its empirical scope and the methodological conditions under which it was conducted. In particular, given the non-experimental and cross-sectional design, the results allow for the identification of statistically significant associations between the valuation of AIA and Decision Making, but they do not establish causal relationships or temporal dynamics between the variables.

Likewise, the evidence is based on perceptions reported by public officials with national-level decision-making responsibilities. One limitation of this approach is the potential for response bias related to participants' differing levels of familiarity with artificial intelligence, which may influence how the evaluated constructs are perceived. This approach is appropriate for understanding the assessments and criteria of decision makers; however, it suggests the value of complementing this perspective with objective indicators of decision-making performance and with observational evidence drawn from real-world public decision contexts.

Although the study incorporates core dimensions of decision making, some of them, such as integrality, transparency, and coherence, still lack fully standardized quantitative metrics in the indexed literature. In this sense, the results provide relevant empirical evidence for a field that is still under consolidation, while also highlighting the opportunity to further advance the validation and harmonization of measures that would enable more direct comparisons across studies and institutional contexts.

### 6.2 Future research

Future research could extend these findings through longitudinal or quasi-experimental designs that allow observation of the stability of the association between AIA and decision making over time and across different levels of institutional adoption.

Future research should also consider the contextual and organizational conditions under which public-sector decisions are made, including situations where decisions are constrained, non-alternative, or influenced by implicit institutional and political factors. In such contexts, the integration of artificial intelligence should be examined in conjunction with human judgment and participatory approaches, as these may enhance the robustness and sustainability of decision-making processes. Additionally, further research could explore how different types of decisions (e.g., strategic, tactical, individual, or collective) may condition the effectiveness and applicability of AI-based decision support systems.

Likewise, it would be appropriate to complement perception-based metrics with objective operational indicators in order to contrast results in real public management contexts.

The survey instrument used in this study, available in both Spanish and English versions, is made available to the scientific community for use and application in other sectors and countries, which would facilitate systematic comparisons and the progressive accumulation of empirical evidence on the evaluated dimensions of decision making.

## Data Availability

The original contributions presented in the study are included in the article/Supplementary material, further inquiries can be directed to the corresponding author.

## Supplementary material

Supplementary File 1 Consistency matrix.

Supplementary File 2 Operationalization matrix.

Supplementary File 3 Spanish survey.

Supplementary File 4 English survey.
