# Supplementary material for: Artificial intelligence agents as advanced decision support systems in public decision-making: evidence from Peru
Source: Front Artif Intell. 2026 May 13;9:1805539. doi: 10.3389/frai.2026.1805539 (PMC13212331; doi:10.3389/frai.2026.1805539)
Supplement: Supplementary File 1 — Consistency matrix. [file Data_Sheet_1.pdf]

Public Decision-Making Assisted by Artificial Intelligence Agents: Quantitative Evidence and Governance Challenges

Research Problems	Research Objectives	Research Hypotheses	Variables	Dimensions	Indicators
What is the association between the valuation of artificial intelligence agents and public officials' decision-making, considering both the overall quality of the decision-making process and its main dimensions, within the context of Peruvian public administration?	To analyze the association between the valuation of artificial intelligence agents and public officials' decision-making, considering both the overall quality of the decision-making process and its main dimensions, within the context of Peruvian public administration.	There is an association between the valuation of artificial intelligence agents and public officials' decision-making, considering both the overall quality of the decision-making process and its main dimensions, within the context of Peruvian public administration.	Artificial Intelligence Agents	Knowledge	General knowledge of the concept of AI agents<br>Familiarity with concrete use examples (chatbots, virtual assistants, etc.)<br>Prior experience interacting with automated or semi-automated systems
				Perceived usefulness	Expectation of improvement in administrative and/or decision-making processes<br>Perceived added value in terms of efficiency or error reduction<br>Potential to automate repetitive tasks and free up valuable time
				Ease of use	Perceived technical complexity for adoption<br>Availability of institutional training or capacity-building resources<br>Level of support (technical, organizational) to implement and maintain AI agents
				Predisposition	Willingness to use or promote the use of AI agents<br>Degree of confidence in the results provided by AI agents<br>Interest in training and/or engaging in AI-related projects
What is the association between the valuation of artificial intelligence agents and the speed of the decision-making process among public officials?	To analyze the association between the valuation of artificial intelligence agents and the speed of the decision-making process among public officials.	There is an association between the valuation of artificial intelligence agents and the speed of the decision-making process among public officials.	Decision Making	Speed	Perception of the speed at which data are processed for decision-making<br>Response capacity in urgent or critical situations<br>Perceived agility in the processing of authorizations or case analysis
What is the association between the valuation of artificial intelligence agents and perceived accuracy in public decision-making?	To analyze the association between the valuation of artificial intelligence agents and perceived accuracy in public decision-making.	There is an association between the valuation of artificial intelligence agents and perceived accuracy in public decision-making.		Accuracy	Perception of the reliability of the information used in decisions<br>Frequency or likelihood of errors in decisions<br>Degree of alignment with available quantitative and qualitative evidence or data
What is the association between the valuation of artificial intelligence agents and the integrality of the decision-making process, understood as the inclusion of multiple sources and perspectives?	To analyze the association between the valuation of artificial intelligence agents and the integrality of the decision-making process, understood as the inclusion of multiple sources and perspectives.	There is an association between the valuation of artificial intelligence agents and the integrality of the decision-making process, understood as the inclusion of multiple sources and perspectives.		Integrality	Diversity of information sources considered in the decision<br>Inclusion of different perspectives (technical, social, economic criteria, etc.)<br>Breadth and depth of the data analyzed
What is the association between the valuation of artificial intelligence agents and the transparency of the public decision-making process?	To analyze the association between the valuation of artificial intelligence agents and the transparency of the public decision-making process.	There is an association between the valuation of artificial intelligence agents and the transparency of the public decision-making process.		Transparency	Clarity in the justification and documentation of decisions<br>Accessible information on the criteria used (traceability)<br>Capacity to provide accountability to oversight bodies or the public
What is the association between the valuation of artificial intelligence agents and the coherence of decisions with institutional regulatory and strategic frameworks?	To analyze the association between the valuation of artificial intelligence agents and the coherence of decisions with institutional regulatory and strategic frameworks.	There is an association between the valuation of artificial intelligence agents and the coherence of decisions with institutional regulatory and strategic frameworks.		Coherence	Alignment of the decision with institutional strategic plans<br>Compliance with existing regulations and policies<br>Contribution to long-term goals (expected impact)
