# Supplementary material for: Artificial intelligence agents as advanced decision support systems in public decision-making: evidence from Peru
Source: Front Artif Intell. 2026 May 13;9:1805539. doi: 10.3389/frai.2026.1805539 (PMC13212331; doi:10.3389/frai.2026.1805539)
Supplement: Supplementary File 2 — Operationalization matrix. [file Data_Sheet_2.pdf]

Public Decision-Making Assisted by Artificial Intelligence Agents: Quantitative Evidence and Governance Challenges

Variable	Conceptual definition	Operational definition	Dimensions	Indicators	#	Items
Artificial Intelligence Agents	Artificial intelligence agents (AIAs) have been defined as “systems capable of making decisions, interacting with their environment, and performing tasks autonomously” (Jovanović & Campbell, 2025).	The variable has been broken down and analyzed across four dimensions and 12 indicators, using a survey of 12 statements in the AIA variable that measures responses on the Likert scale.	Knowledge	General knowledge of the concept of AI agents	1	I am familiar with the definition and functions of artificial intelligence agents.
				Familiarity with concrete use examples (chatbots, virtual assistants, etc.)	2	I am familiar with examples of artificial intelligence agents, such as chatbots or virtual assistants, that could be applied in my institution.
				Prior experience interacting with automated or semi-automated systems	3	I have interacted satisfactorily with automatic or semi-automatic AI-based systems, either within or outside my work environment.
			Perceived usefulness	Expectation of improvement in administrative and/or decision-making processes	4	I perceive that the use of artificial intelligence agents can improve administrative and/or decision-making processes in my institution.
				Perceived added value in terms of efficiency or error reduction	5	I perceive that artificial intelligence agents are useful for reducing potential errors in administrative tasks.
				Potential to automate repetitive tasks and free up valuable time	6	I perceive significant added value in the use of artificial intelligence agents to improve decision-making.
			Ease of use	Perceived technical complexity for adoption	7	I believe that artificial intelligence agents can be learned and easily managed by the staff of my institution.
				Availability of institutional training or capacity-building resources	8	I believe that my institution provides (or would provide) the necessary training for the proper implementation of artificial intelligence agents.
				Level of support (technical, organizational) to implement and maintain AI agents	9	I have sufficient technical and organizational support to adopt artificial intelligence agents in my work area.
			Predisposition	Willingness to use or promote the use of AI agents	10	I am interested in participating in initiatives that involve the implementation of artificial intelligence agents.
				Degree of confidence in the results provided by AI agents	11	I trust the information and recommendations that artificial intelligence agents can provide for my daily work tasks.
				Interest in training and/or engaging in AI-related projects	12	I have a positive attitude toward the adoption of artificial intelligence agents in my institution.
Decision Making	Decision-making in the public sector is defined as the set of processes through which government officials and organizations adopt resolutions that impact citizens (Bolton, 2024).	The variable has been broken down and analyzed across five dimensions and 15 indicators, using a survey of 15 statements in the decision making variable that measures responses on the Likert scale.	Speed	Perception of the speed at which data are processed for decision-making	13	I believe that artificial intelligence agents can significantly speed up data collection for decision-making.
				Response capacity in urgent or critical situations	14	I believe that artificial intelligence agents improve response capacity in urgent situations.
				Perceived agility in the processing of authorizations or case analysis	15	I perceive that artificial intelligence agents streamline case analysis and enable faster solution development.
			Accuracy	Perception of the reliability of the information used in decisions	16	I trust that artificial intelligence agents provide reliable and accurate information in decision-making processes.
				Frequency or likelihood of errors in decisions	17	I believe that artificial intelligence agents reduce the likelihood of errors when selecting and analyzing information.
				Degree of alignment with available quantitative and qualitative evidence or data	18	I believe that artificial intelligence agents facilitate the use of relevant and up-to-date data to support decision-making.
			Integrality	Diversity of information sources considered in the decision	19	I perceive that artificial intelligence agents allow data from multiple sources to be gathered simultaneously, broadening the understanding of the problem.
				Inclusion of different perspectives (technical, social, economic criteria, etc.)	20	I believe that artificial intelligence agents promote the inclusion of multiple perspectives (technical, economic, social) in the decision-making process.
				Breadth and depth of the data analyzed	21	I view artificial intelligence agents as an opportunity to achieve a more comprehensive and in-depth perspective in public management.
			Transparency	Clarity in the justification and documentation of decisions	22	I believe that the application of artificial intelligence agents promotes the traceability of data used in decision-making.
				Accessible information on the criteria used (traceability)	23	I believe that artificial intelligence agents facilitate clear explanations of how a decision or policy outcome was reached.
				Capacity to provide accountability to oversight bodies or the public	24	I believe that the use of artificial intelligence agents strengthens accountability toward oversight bodies and the public.
			Coherence	Alignment of the decision with institutional strategic plans	25	I perceive that artificial intelligence agents help align decisions with institutional strategic objectives.
				Compliance with existing regulations and policies	26	I believe that artificial intelligence agents contribute to compliance with existing regulations and policies.
				Contribution to long-term goals (expected impact)	27	I believe that, with the support of artificial intelligence agents, decisions are better aligned with long-term planning and goals.
