# Supplementary material for: Artificial intelligence agents as advanced decision support systems in public decision-making: evidence from Peru
Source: Front Artif Intell. 2026 May 13;9:1805539. doi: 10.3389/frai.2026.1805539 (PMC13212331; doi:10.3389/frai.2026.1805539)
Supplement: Supplementary File 3 — Spanish survey. [file Data_Sheet_3.pdf]

Survey– Artificial Intelligence Agents as Advanced Decision Support Systems in Public Decision-Making: Evidence from Peru

MD = Muy en desacuerdo (1) / **ED** = En desacuerdo (2) / **I** = Indeciso (3) / **DA** = De acuerdo (4) / **MA** = Muy de acuerdo (5)

ITEMS	ESCALE				
**Variable: Agentes de Inteligencia Artificial**	MD	ED	I	DA	MA
**Dimension 1: Conocimiento**					
1. Estoy familiarizado(a) con la definición y funciones de los agentes de Inteligencia Artificial.					
2. Tengo claros ejemplos de agentes de IA, como chatbots o asistentes virtuales, que podrían aplicarse en mi institución.					
3. He interactuado satisfactoriamente con sistemas automáticos o semiautomáticos basados en IA fuera o dentro de mi entorno laboral.					
**Dimension 2: Percepción de Utilidad**					
4. Percibo que el uso de agentes de IA puede mejorar los procesos administrativos y/o de toma de decisiones en mi institución.					
5. Percibo que los Agentes de Inteligencia Artificial son útiles para reducir posibles errores en las tareas administrativas.					
6. Veo un gran valor agregado en el uso de agentes de IA para optimizar la toma de decisiones.					
**Dimension 3: Facilidad de Uso**					
7. Confío en que los agentes de IA pueden ser aprendidos y manejados con facilidad por el personal de mi institución.					
8. Siento que mi institución provee (o proveería) la capacitación necesaria para la correcta implementación de agentes de IA.					
9. Cuento con el respaldo técnico y organizacional suficiente para adoptar agentes de IA en mi área de trabajo.					
**Dimension 4: Predisposición**					
10. Me interesa participar en iniciativas que incluyan la implementación de agentes de IA.					
11. Confío en la información y las sugerencias que pueden brindar los agentes de IA para mis labores diarias.					
12. Tengo una actitud positiva hacia la adopción de agentes de IA en mi institución.					

Survey– Artificial Intelligence Agents as Advanced Decision Support Systems in Public Decision-Making: Evidence from Peru

MD = Muy en desacuerdo (1) / **ED** = En desacuerdo (2) / **I** = Indeciso (3) / **DA** = De acuerdo (4) / **MA** = Muy de acuerdo (5)

ITEMS	SCALE				
**Variable: Toma de Decisiones**	SD	D	U	A	SA
**Dimension 1: Rapidez**					
1. Considero que los agentes de IA pueden acelerar de forma notable la recopilación de datos para decidir.					
2. Estoy convencido(a) de que los agentes de IA mejoran la capacidad de respuesta ante situaciones urgentes.					
3. Percibo que los agentes de IA agilizan el análisis de casos y permiten formular soluciones más rápidas.					
**Dimension 2: Exactitud**					
4. Confío en que los agentes de IA proporcionan información confiable y precisa en los procesos de decisión.					
5. Creo que los agentes de IA disminuyen la probabilidad de errores al seleccionar y analizar información.					
6. Estoy seguro(a) de que los agentes de IA facilitan la utilización de datos relevantes y actualizados para fundamentar las decisiones.					
**Dimension 3: Integralidad**					
7. Percibo que los agentes de IA permiten reunir datos de diversas fuentes simultáneamente, ampliando la visión del problema.					
8. Estoy convencido(a) de que los agentes de IA fomentan la inclusión de múltiples perspectivas (técnicas, económicas, sociales) en el proceso de decisión.					
9. Veo en los agentes de IA una oportunidad para tener una visión más completa y profunda en la gestión pública.					
**Dimension 4: Transparencia**					
10. Considero que la aplicación de agentes de IA promueve la trazabilidad de los datos empleados al tomar decisiones.					
11. Creo que los agentes de IA facilitan que se explique con claridad cómo se llegó a una resolución o política.					
12. Estoy seguro(a) de que el uso de agentes de IA refuerza la rendición de cuentas ante entidades de control y la ciudadanía.					
**Dimension 5: Coherencia**					
13. Percibo que los agentes de IA ayudan a alinear las decisiones con los objetivos estratégicos institucionales.					
14. Estoy convencido(a) de que los agentes de IA contribuyen al cumplimiento de las normas y políticas vigentes.					
15. Considero que, gracias a los agentes de IA, las decisiones se ajustan mejor a la planificación y metas de largo plazo.					
