# Supplementary material for: Artificial intelligence agents as advanced decision support systems in public decision-making: evidence from Peru
Source: Front Artif Intell. 2026 May 13;9:1805539. doi: 10.3389/frai.2026.1805539 (PMC13212331; doi:10.3389/frai.2026.1805539)
Supplement: Supplementary File 4 — English survey. [file Data_Sheet_4.pdf]

Survey - Artificial Intelligence Agents as Advanced Decision Support Systems in Public Decision-Making: Evidence from Peru

SD = Strongly disagre (1) / **D** = Disagre (2) / **U** = Undecided (3) / **A** = Agre (4) / **SA** = Strongly agre (5)

ITEMS	SCALE				
**Variable: Artificial Intelligence Agents**	SD	D	U	A	SA
**Dimension 1: Knowledge**					
1. I am familiar with the definition and functions of artificial intelligence agents.					
2. I am familiar with examples of artificial intelligence agents, such as chatbots or virtual assistants, that could be applied in my institution.					
3. I have interacted satisfactorily with automatic or semi-automatic AI-based systems, either within or outside my work environment.					
**Dimension 2: Perceived usefulness**					
4. I perceive that the use of artificial intelligence agents can improve administrative and/or decision-making processes in my institution.					
5. I perceive that artificial intelligence agents are useful for reducing potential errors in administrative tasks.					
6. I perceive significant added value in the use of artificial intelligence agents to improve decision-making.					
**Dimension 3: Perceived usefulness**					
7. I believe that artificial intelligence agents can be learned and easily managed by the staff of my institution.					
8. I believe that my institution provides (or would provide) the necessary training for the proper implementation of artificial intelligence agents.					
9. I have sufficient technical and organizational support to adopt artificial intelligence agents in my work area.					
**Dimension 4: Predisposition**					
10. I am interested in participating in initiatives that involve the implementation of artificial intelligence agents.					
11. I trust the information and recommendations that artificial intelligence agents can provide for my daily work tasks.					
12. I have a positive attitude toward the adoption of artificial intelligence agents in my institution.					

Survey - Artificial Intelligence Agents as Advanced Decision Support Systems in Public Decision-Making: Evidence from Peru

SD = Strongly disagre (1) / **D** = Disagre (2) / **U** = Undecided (3) / **A** = Agre (4) / **SA** = Strongly agre (5)

ITEMS	SCALE				
**Variable: Decision Making**	SD	D	U	A	SA
**Dimension 1: Speed**					
1. I believe that artificial intelligence agents can significantly speed up data collection for decision-making.					
2. I believe that artificial intelligence agents improve response capacity in urgent situations.					
3. I perceive that artificial intelligence agents streamline case analysis and enable faster solution development.					
**Dimension 2: Accuracy**					
4. I trust that artificial intelligence agents provide reliable and accurate information in decision-making processes.					
5. I believe that artificial intelligence agents reduce the likelihood of errors when selecting and analyzing information.					
6. I believe that artificial intelligence agents facilitate the use of relevant and up-to-date data to support decision-making.					
**Dimension 3: Integrality**					
7. I perceive that artificial intelligence agents allow data from multiple sources to be gathered simultaneously, broadening the understanding of the problem.					
8. I believe that artificial intelligence agents promote the inclusion of multiple perspectives (technical, economic, social) in the decision-making process.					
9. I view artificial intelligence agents as an opportunity to achieve a more comprehensive and in-depth perspective in public management.					
**Dimension 4: Transparency**					
10. I believe that the application of artificial intelligence agents promotes the traceability of data used in decision-making.					
11. I believe that artificial intelligence agents facilitate clear explanations of how a decision or policy outcome was reached.					
12. I believe that the use of artificial intelligence agents strengthens accountability toward oversight bodies and the public.					
**Dimension 5: Coherence**					
13. I perceive that artificial intelligence agents help align decisions with institutional strategic objectives.					
14. I believe that artificial intelligence agents contribute to compliance with existing regulations and policies.					
15. I believe that, with the support of artificial intelligence agents, decisions are better aligned with long-term planning and goals.					
